# Atlanta Medical Society

**Published:** 1856-12

**Authors:** 


### EXTRACTS FROM THE RECORDS OF THE ATLANTA MEDICAL SOCIETY.

REPORTED BY THE SECRETARY PRO TEM.

*Nov. 6th.* 1856.

Dr. M. H. Oliver reported a case of coxalgia of some eighteen months standing, that had recently been put upon the internal use of iodide of potassium and sarsaparilla, with the application of tincture of iodine to the hip-joint, under which treatment there had been marked improvement.

During the discussion of the regular question, "Can we have Typhoid Fever with Intermittent Symptoms," Dr. Oliver remarked, that the only case of typhoid fever he had ever treated, manifested marked intermittent symptoms in the commencement, continuing four or five days, then assuming the ordinary course of continued fever. He was sustained in his opinion as to the character of the disease, by the opinion of several of the prominent physicians of the city.

Dr. Wilson was clearly of the opinion, that we do have typhoid fever occurring in this locality, with the ordinary symptoms of intermittent fever, during the first week, at least so far as a regular freedom from fever at stated periods, and periodical exacerbations—he referred particularly to a case of comparatively recent occurrence, in which he had verified the correctness of his diagnosis, by a postmortem examination. There was a periodical freedom from fever every morning, and a daily exacerbation, with pain in the lumbar region, &c., and the periodical symptoms were altogether so decided, that he had treated the case with quinine, without, however, deriving any benefit from the remedy. These symptoms continued for about six days, and the ordinary continued course of fever then supervened, and with such violence as to have resulted in a fatal termination about the sixteenth day from the commencement of the attack. The postmortem revealed ulceration of Peyer's glands, with perforation of the bowel.

Dr. J. G. Westmoreland remarked, that he had never witnessed a case of typhoid fever with lumbar pain, and believed it to be very unusual; much more rare, even from a persistent recumbent posture, than in other diseases. If the pain in the lumbar region were very decided, he would be inclined to doubt whether a case were really typhoid fever, notwithstanding strong testimony in other particulars, especially if attended with periodical symptoms.

Dr. Westmoreland remarked, however, that he believed that we might have, and do have, distinct periodical symptoms in typhoid fever in its early stages. He had witnessed this in cases occurring in connection with others of a continued character; he also remarked, that no man, in his opinion, could decide, in reference to whether a case is typhoid fever in its commencement, or during the first week, without, what he considered, the *pathognomonic* symptom; tenderness in the region of the medulla oblongata.

Dr. Wilson stated, that he had been *feeling* for this symptom about ten months, without being able to find it uniformly present.

Dr. Coe remarked that, as to whether the disease does present itself under the form of periodicity, or of an intermittent, or remittent character, he was not prepared to decide. The different opinions in the profession as to its cause, the nature of the morbific agent, its locality, the tissue in which it is located, all render it more difficult to decide as to the true nature of the disease; but that there is frequently a partial remission, if not an entire abatement of the fever every morning, which continues even until evening, is unquestionable, especially in sporadic cases, for five or six days after the commencement of the disease; and in some instances, at least, we may find it more difficult to form a correct diagnosis during a few of the first days of the disease, than has been supposed by some, for he knew of no pathognomonic symptom of the disease, especially in the forming stage.

It is true, that usually, its mode of access is much more gradual (yet, not always so) than intermittent or remittent fever. We have chills, or at least, chilly sensations, fever, headache, occurring every evening, which are all absent in the forepart of the day. The appearance of the tongue, at this stage, differs but little from that presented in remittent fever; it is moist, thinly coated, and, in many cases, not even red at the tip or edges; but in most cases is tremulous when protruded. We also have aching of the back and inferior extremities, which, also occurs in remittent and intermittent fever.

It is very true, that after a few of the first days, typhoid fever becomes well marked, and cannot well be mistaken by any practitioner who is acquainted with his profession. But it is of vast importance to our patient that we distinguish the disease at first, for, if we mistake it for remittent or intermittent fever, and treat it as such, with quinine and calomel, we will not benefit our patient in the least, but do decided injury, and be in great danger of destroying the subject; whilst on the other hand, if we mistake it for typhoid (the safest of the two), and treat it accordingly, we let our patient lie and suffer, and the repeated excitement allowed to continue, produces local disease, which is not relieved except by a long and tedious process, if it do not in the end prove fatal.

So, if we have remittent or intermittent *typhoid fever*, it is all-important that we should be able to distinguish it from our common *periodical* fevers, which come under the head of *remittent* or *intermittent* proper. As to the pathognomonic symptom alluded to by Dr. Westmoreland—the tenderness at the juncture of the spine with the occiput—he was not prepared to decide, as his opportunity for investigation had been too limited, since his attention was directed to that point, to enable him to venture any decided opinion. In one well marked case of typhoid fever which he had examined since, he failed to detect the tenderness, which might have been attributable to the period of the disease, or his want of skill in the investigation. He concluded with the remark, that if it could be established that typhoid fever affects, specifically, this portion of the nervous structure, he should be much more favorably disposed to the view entertained by some—that this fever might present periodicity, than he was at present.

Dr. Logan remarked, that he had been very incredulous in reference to periodical symptoms in typhoid fever until recently he had been called to treat several cases, presenting, to a great extent, the ordinary symptoms of periodical fever, for a number of days, in the first stage, which had afterwards, assumed the usual appearance, and pursued the regular course of *continued* fever. He, however, thought we ought to be very cautious how we adopted views, so entirely opposed to the usual doctrine upon this subject, and should be certain of being well fortified, by evidence of a decided character.

Dr. Westmoreland called attention to the fact, that in typhoid fever, we had usually an active perspiration during the fever, without the reduction of excitement, while in periodical fever proper, the perspiration occurred with the subsidence of the fever, constituting a regular *stage.*

The following resolution, offered by Dr. Coe, was adopted by the Society: That the members of this Society be requested to note particularly the symptoms of every case of disease, which they may suppose to bear any resemblance to typhoid fever, and report the same.

[The subject here presented is certainly one of very considerable importance; for, as suggested in the discussion, the character of the *diagnosis in the first stages*, will *radically* affect the treatment, and in all probability, the issue of the case. The writer has been accustomed to treat typhoid fever for a number of years, previous to a settlement in this city; and while he has some indistinct recollection of a single case of finally well developed typhoid fever, presenting the usual symptoms of remittent fever, during the first few days of the attack, it is an exceptional case in his experience, until very recently, he has met with some cases, which, so far as the weight of evidence (save a postmortem) can go to establish the nature of the disease, were *decided* to be typhoid fever; yet, for some days, perhaps a week, the fever was clearly of a periodical character; not attended by regular chills, or perhaps, an entire intermission, but by a distinct daily remission.

The occasional manifestation of a periodical element, has been recognized in this disease since the publication of Dr. Bartlett's Treatise on Fevers, though we should hardly have been prepared to expect it in a locality where the occurrence of malarious fevers is so rare; so much so, indeed, that it has been a matter of discussion, among the medical men here, whether they existed at all—that we do, however, occasionally have them originating here is now a settled point.

It is certainly a desideratum to be able to determine with more accuracy, whether a case be really typhoid fever in the *commencement*. It is admitted upon all hands, that there is no special difficulty after the disease has been fully developed. In reference to the diagnostic symptom to which so much importance is attached by Dr. J. G. Westmoreland, we can only remark, that while there may never be a case of typhoid fever without an affection of the medulla oblongata, and the external evidence of that affection, there are disturbances and lesions of that nervous centre without typhoid fever; and we cannot, therefore, attach the importance to it that he seems to think it deserves—so far at least, as to be able to determine by its presence, that we have the disease under discussion. That this point may be one of those specifically affected in the disease we are not prepared to deny, nor to assert that its discovery is not an advance in pathology, or of practical benefit in the management of this obscure and troublesome affection, but that it deserves to be looked upon as an infallible diagnostic mark, we very much question.—Editor.]

